# Data integration enables global biodiversity synthesis

**DOI:** 10.1073/pnas.2018093118

**Published:** 2021-02-01

**Authors:** J. Mason Heberling, Joseph T. Miller, Daniel Noesgaard, Scott B. Weingart, Dmitry Schigel

**Affiliations:** ^a^Section of Botany, Carnegie Museum of Natural History, Pittsburgh, PA 15213;; ^b^Global Biodiversity Information Facility, Secretariat, DK-2100 Copenhagen Ø, Denmark;; ^c^Digital Humanities Program, University Libraries, Carnegie Mellon University, Pittsburgh, PA 15213

**Keywords:** biodiversity informatics, community science, Global Biodiversity Information Facility (GBIF), biological collections, scientometrics

## Abstract

The accessibility of global biodiversity information has surged in the past two decades, notably through widespread funding initiatives for museum specimen digitization and emergence of large-scale public participation in community science. Effective use of these data requires the integration of disconnected datasets, but the scientific impacts of consolidated biodiversity data networks have not yet been quantified. To determine whether data integration enables novel research, we carried out a quantitative text analysis and bibliographic synthesis of >4,000 studies published from 2003 to 2019 that use data mediated by the world’s largest biodiversity data network, the Global Biodiversity Information Facility (GBIF). Data available through GBIF increased 12-fold since 2007, a trend matched by global data use with roughly two publications using GBIF-mediated data per day in 2019. Data-use patterns were diverse by authorship, geographic extent, taxonomic group, and dataset type. Despite facilitating global authorship, legacies of colonial science remain. Studies involving species distribution modeling were most prevalent (31% of literature surveyed) but recently shifted in focus from theory to application. Topic prevalence was stable across the 17-y period for some research areas (e.g., macroecology), yet other topics proportionately declined (e.g., taxonomy) or increased (e.g., species interactions, disease). Although centered on biological subfields, GBIF-enabled research extends surprisingly across all major scientific disciplines. Biodiversity data mobilization through global data aggregation has enabled basic and applied research use at temporal, spatial, and taxonomic scales otherwise not possible, launching biodiversity sciences into a new era.

## Significance

As anthropogenic impacts to Earth systems accelerate, biodiversity knowledge integration is urgently required to support responses to underpin a sustainable future. Consolidating information from disparate sources (e.g., community science programs, museums) and data types (e.g., environmental, biological) can connect the biological sciences across taxonomic, disciplinary, geographical, and socioeconomic boundaries. In an analysis of the research uses of the world’s largest cross-taxon biodiversity data network, we report the emerging roles of open-access data aggregation in the development of increasingly diverse, global research. These results indicate a new biodiversity science landscape centered on big data integration, informing ongoing initiatives and the strategic prioritization of biodiversity data aggregation across diverse knowledge domains, including environmental sciences and policy, evolutionary biology, conservation, and human health.

As we enter the sixth mass extinction (1, 2), effective Earth stewardship requires high volumes of biodiversity data across scales (2, 3), provided in openly accessible, verifiable, and usable formats (i.e., FAIR Data Principles [findability, accessibility, interoperability, reusablility] that serve as best practice guidelines for data providers and publishers) (4). However, the necessary infrastructure for the integration of disparate data poses significant informatic and social challenges (5). Efforts over the past 20 y have led to global data networks that aggregate biodiversity datasets into consolidated data portals (6), providing online access to genetic (7), phenotypic (8), ecological (9), and occurrence (10) information at the level of individuals to biomes. Among those is the world’s single largest biodiversity data portal maintained by the Global Biodiversity Information Facility (GBIF; http://gbif.org), an intergovernmental research infrastructure providing open access to biodiversity data and resources for data publishing and use. Formed at the start of the “big data” concept (11), GBIF was established with a strong museum specimen-based focus (6), and, while maintaining these roots, has since evolved to include many new data sources (12). Given the technological, analytical, and conceptual advances made since GBIF was formed in 2001 (11), a comprehensive analysis and review of aggregated biodiversity data use is now needed to quantify the scientific impacts of data mobilization and promote the continued development for the next generation of biodiversity-related research.

Over the past 20 y, biodiversity research has been transformed by a big data revolution (5, 11, 13). The digitization of previously inaccessible data (“dark data,” ref. 14) and rapid new data creation through public participation in research (hereafter referred to as “community science”) (15) has led to unprecedented biodiversity data mobilization, much of which is available via GBIF. Since 2011 alone, the US National Science Foundation funded program iDigBio has mobilized more than 120 million specimens held in US institutions—with concurrent efforts continuing in parallel across the world (16). Likewise, new observation-based records collected through community science platforms have proliferated, with pioneering programs such as eBird [>700 million occurrences (17)] http://observation.org [>39 million occurrences (18)], and iNaturalist [>18 million occurrences (19)], outpacing museum specimen digitization by orders of magnitude (12). A major challenge to data use, however, is the integration of disparate datasets for efficient and reliable research use (6, 20–22).

Recent studies have focused on spatial, taxonomic, and temporal data gaps of the GBIF-mediated data themselves (12, 23–29), but the scientific impacts and patterns of GBIF-mediated data use have not been quantified. Concomitant with GBIF growth, the development of species distribution modeling (SDM) statistical techniques (30) and increased natural history collection digitization (16) suggests GBIF data use may be strongly directed toward research in species distributions and taxonomy/systematics. However, aside from speculation, the scope, patterns, and novelty of research stimulated by GBIF-available data remains largely unknown.

Leveraging a dataset of >4,000 studies that rely upon GBIF-mediated data, we provide a comprehensive analysis of data use patterns of a global biodiversity data network. We broadly asked: 1) Is biodiversity data growth matched by research use? 2) Are certain data types used more and by whom? And especially: 3) Do certain research topics dominate GBIF-mediated studies and have they changed through time?

To quantify the major research themes and their temporal trends in the GBIF-enabled literature (i.e., studies that rely upon GBIF-mediated data), we performed a computational text analysis called topic modeling, also known as automated content analysis. Topic models use machine learning methods to classify texts according to probability distributions of word cooccurrence within texts and among the entire corpus (i.e., all texts analyzed). Initially developed in the social sciences and humanities (31), topic modeling has gained traction in biology to synthesize large volumes of literature (32). Here, we used a variant called structural topic modeling (STM) (33) derived from the widely used latent Dirichelet allocation (LDA) topic model approach (34). STM is an unsupervised, mixed-membership model, meaning that topics emerge inductively (i.e., no a priori assignment of topics by researcher), and each text can be classified to multiple topics. Each document is represented as a vector of topic proportions according to fractions of words assigned to a given topic. We combined topic modeling, science mapping (35), and traditional review to quantify the scope, trends, and broader thematic landscape of GBIF-enabled data use.

## Results

### Biodiversity Data Availability and Use Has Increased.

**Fig. 1.**
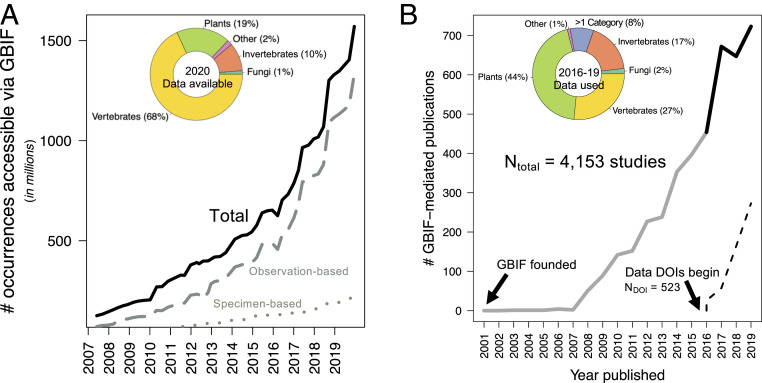
Growth over time of the biodiversity occurrence data accessible via the Global Biodiversity Information Facility (GBIF) (*A*) and peer-reviewed articles using these data (*B*). Occurrence data (solid line in *A*) is further broken into observation-based records (dashed) and museum specimen-based records (dotted). Pie charts illustrate proportional taxonomic representation in GBIF datasets as of July 2020 (*A*) and corresponding representation of data use in recently published articles (2016 to 2019; solid black line) (*B*). “Other” refers to organismal groups not included in other categories (*A* and *B*). “>1 category” refers to data use of multiple organismal groups (*B*). Citable digital object identifiers (DOIs) were provided with each GBIF data download since 2016 (dash line in *B*).

Data available through GBIF have surged in the past decade, growing by 1,150% since 2007 (2007: 125 million; 2020: 1.6 billion occurrence records; Fig. 1*A*). Both observation- and specimen-based (i.e., those linked to physical vouchers) (12) records have increased. The overall increase was strongly driven by the expansion of public participation and observation-based datasets. Community science-generated datasets (i.e., data collected primarily by volunteers, frequently called “citizen science” or “public participation in research”) (15) only accounted for 11% of occurrence data in 2007, yet account for 65% of data in 2020. Specimen-based occurrences comprised 14% of GBIF-mediated data in 2020 (85% observation based, 1% not reported by data publisher), a decrease from 25% in 2007. Despite numerical dominance of observation-based data, specimen-based data notably increased through museum digitization efforts, with 187.7 million specimens newly mobilized from 2007 to 2020 (sixfold increase).

Research use of GBIF-mediated data has similarly risen in the past decade, with 723 peer-reviewed studies published in 2019 alone compared to 148 studies published cumulatively from 2003 to 2009 (Fig. 1*B*). In 2016, GBIF began issuing digital object identifiers (DOIs) with each data download to effectively track data use. Best data practices include citing data DOI(s) in publications. Though increasing, only a minority of authors cite a DOI (38% of studies in 2019). Of the 520 studies with a GBIF-specific data DOI, the number of records cited per study range from single occurrence points to 1.6 billion (median = 9,071; interquartile range = 699 to 227,302). Of the 26,046 separate datasets in GBIF with at least 1 study citing data, median citation rate is 11 studies per dataset (highest: 713). Community science datasets tend to have more citations (Median_community_ _science_ = 13; Median_noncommunity_ _science_ = 8; Wilcoxon rank sum test, W = 6,211,175, *P* < 0.001), which is perhaps unsurprising, as larger datasets tend to have more citations (Spearman’s correlation, rho = 0.39, df = 32,635, *P* < 0.001). However, when controlling for dataset size, the opposite is true (dataset citations scaled per 100 occurrence records: Median_community_ _science_ = 0.1; Median_noncommunity_ _science_ = 3; W = 11,514,982, *P* < 0.001).

Taxonomic discrepancies exist between data availability and data use. For example, vertebrate taxa account for 68% of current GBIF-available data (Fig. 1*A*), yet proportionally fewer studies use these data (27% of 2,496 publications from 2016 to 2019; Fig. 1*B*). Conversely, plants are the most common use of GBIF-mediated data, representing nearly half of recent studies (44%) but only comprise 19% of GBIF-available data.

### Data Integration Facilitates Global Research and Access.

**Fig. 2.**
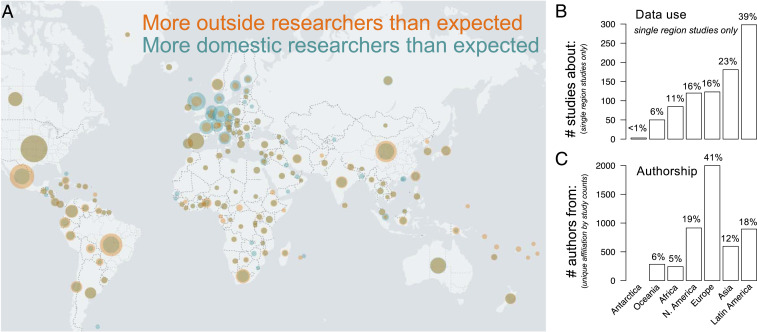
Geography of GBIF data use and authorship. World map (*A*) highlights disparities between country-level biodiversity data use and author affiliation. The map overlays two normalized datasets: orange circles indicate country-level biodiversity data use, and teal circles indicate country-level author affiliations. Circle sizes are proportional to the maximum value in each dataset. Researcher affiliation (teal) is overlaid atop research coverage (orange), mixing to form brown where they overlap. Wider teal rings indicate disproportionately higher number of researchers than research specific to that country (e.g., United Kingdom), whereas wider orange rings (e.g., Mexico) indicate the opposite. Brown circles with no external rings indicate a proportionally similar number of studies about a given country to authors from a given country (e.g., United States). Bar charts show the corresponding frequency of studies published in 2016 to 2019 about a specific region, excluding global studies (*B*) and the frequency of authorship from each region (*C*; unique country-level affiliation by study counts). GBIF regions follow ref. 63.

The global representation of GBIF-mediated data is reflected in research use, with 69% of recent studies (2016 to 2019) spanning more than one continent. However, geographic patterns of research and researcher affiliation are nonrandom. Of those studies focusing on biodiversity at the country- or continental-scale, strong geographic asymmetries exist between author affiliation and the study area (Fig. 2). These studies tend to focus on Latin American biodiversity (39% of 773 single region studies published 2016 to 2019; Fig. 2*A*), whereas most authors are affiliated with European institutions (41% of 4,933 unique author affiliation by study combinations between 2016 and 2019; Fig. 2*B*). A total of 58 of these 733 studies were published entirely by authors affiliated outside the study region. Country-level data use and authorship reveal strong biases—European countries tend to have more researchers than expected based on region-level studies, whereas proportionally more studies on the biodiversity of Mexico, Brazil, and China were published than expected based on the number of authors from those countries (Fig. 2*A*). Authorship biases are similar for studies of global extent (*SI Appendix*, Fig. S5).

### GBIF-Mediated Data Use Is Conceptually Diverse and Temporally Dynamic.

**Fig. 3.**
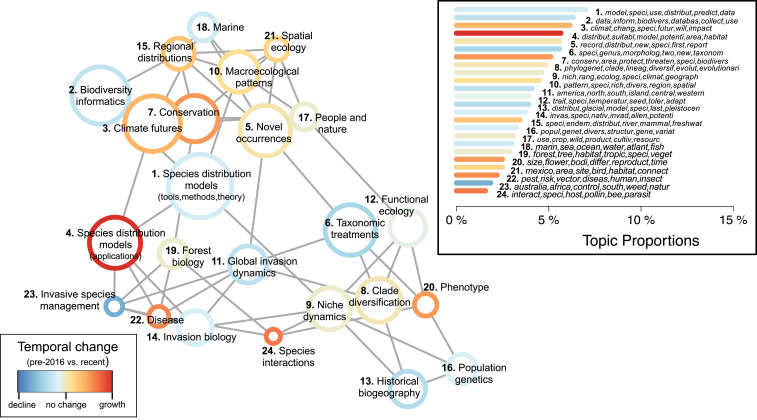
Structural topic model results from 4,035 studies that used GBIF-mediated data published from 2003 to 2019. Topic correlations network visualizes quantitative associations between topics (nodes), with topics near each other and connected by a gray line more likely to appear together in a given study. Node color denotes the relative change in prevalence over time within each topic, comparing topic prevalence in earlier studies (2003 to 2015) to those recently published (2016 to 2019). Node sizes are proportional to overall topic proportions. Network graphed using the Fruchterman–Reingold algorithm. (*Inset*) Bar chart of topic proportions across all years, indicating the percentage of the total corpus that belongs to each topic, with topic numbers corresponding to topic names in network graph and bar color corresponding to temporal change. The top six words by probability associated with each topic are given in italics (*SI Appendix*, Table S1).

Computational text analysis of 4,035 studies from 2003 to 2019 resulted in 24 major topics, each defined by an associated set of high probability words in article titles, abstracts, and keywords (Fig. 3, *Inset*; *SI Appendix*, Table S1). As an unsupervised approach, structural topic model results included a diverse set of topics that emerged without a priori classifications, including application-based (e.g., topic 7, conservation), conceptually based (e.g., topic 20, phenotype), methods-based (e.g., topic 2, biodiversity informatics), and taxonomic/biome-focused (e.g., topic 18, marine biology) topics. No single topic dominated the GBIF-mediated literature. Species distribution modeling methods was the most prevalent (topic 1; 7% of all text analyzed) and species interactions was as the least prevalent (topic 24; 2% of all text analyzed).

We used correlation network analysis to visualize research topic clusters (Fig. 3). Related topics are those that comprise word sets that are shared within and across studies. Topics relating to conservation, biodiversity data use and access, and macroecological patterns clustered together (i.e., upper portion of Fig. 3), with topics relating to discrete concepts of phylogenetic and population-level variation and interactions clustered together (e.g., topic 12 functional ecology; topic 8, clade diversification). Accounting for 31% of the literature, the top five most prevalent topics relate to aspects of SDM use and theory, including all aspects of SDM tools, development, application, and mostly studies predicting species distributions under future climate scenarios. Taxonomic treatments (topic 6) links novel species occurrences (topic 5) and molecular and morphometric topic areas. Interestingly, disease-related topic (topic 22) clusters with invasion biology-related topics (topics 11, 14, and 23).

GBIF research-use areas were not static, with some topics showing marked decline in relative prevalence as others become more common through time (Fig. 3). We compared relative differences in overall topic prevalence through time by comparing studies published from 2016 to 2019, a recent period of rapid growth in GBIF-mediated literature when data DOIs began (62% of analyzed studies), to those published before 2016. Although among the most prevalent topics across all years, the conceptual implementation of SDMs has shifted from theoretical and analytical tool development to application of SDMs. The development of SDM-related tools (topic 1) exhibited a modest decline (−10%), while studies directly applying SDMs toward applied questions increased by 48%, the largest relative increase of all topics. Similarly, closely related to SDMs and conservation topics, climate futures (topic 3) increased by 18%. Likewise, biodiversity informatics (topic 2), including solutions to big data use, access, and aggregation, decreased by 15%. Aside from SDMs, macroecology-related topics, including spatial ecology and large-scale diversity patterns, remained relatively stable. Though accounting for relatively fewer studies overall, emerging topics include species interactions, phenotype, and disease (relative increase by 32%, 25%, and 28%, respectively). Invasive species management (topic 23) showed the largest relative decrease of any topic (−33%). Taxonomic treatments (topic 6) exhibited a relative decrease of 21%. Despite these emerging trends in proportional growth within topics, overall topic ranks (Fig. 3, *Inset*) remained relatively stable between time periods indicating no topic has disappeared or emerged between time periods.

### GBIF-Mediated Data Spans Disciplinary Boundaries.

**Fig. 4.**
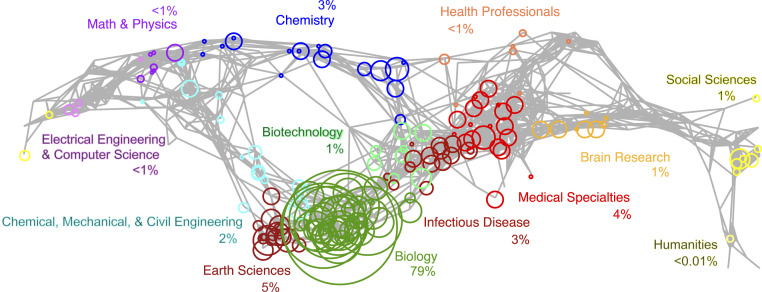
The GBIF map of science, visualizing the network of interdisciplinary knowledge facilitated through GBIF-mediated data in the context of a broader research landscape. The reference base map (gray lines), the UCSD map of science (36), displays a network of >25,000 journals classified across 554 subdisciplines (nodes), grouped into 13 primary disciplines (colors). Circles illustrate GBIF-mediated studies (2003–2019) centered on subdiscipline node assignments with circle size proportion to number of studies. Note that only GBIF-mediated studies published in journals in UCSD map of science are included (2,810 articles, 548 journals). Map is a 2D projection of a spherical 3D layout (i.e., the right and left of map connect) and produced using the *Sci2 Tool* (61).

We summarized the current and potential future use space of GBIF-mediated data through science mapping to visualize literature sets in a broader research landscape (36). GBIF-mediated studies were mapped onto a widely used scientific base map consisting of a network of subdisciplines based on topical clustering of journals (36). GBIF-enabled studies were published in 1,062 journals (746 in 2016 to 2019 alone), of which 30% were open access at time of publication (38% in 2016 to 2019; *SI Appendix*, Fig. S6). The resulting GBIF map of science illustrates cross-disciplinary breadth, with all 13 primary disciplinary categories represented, while also indicating where in the scientific research landscape GBIF-mediated data have not yet been widely applied (Fig. 4). With 10 of the 13 major disciplines each represented by <100 studies, the GBIF-mediated literature is centered on biology-related subdisciplines (79% of mapped studies).

## Discussion

Although open access to large volumes of biodiversity data serves as a logical step toward biodiversity information synthesis, the real value of big data is in its use, not volume. We analyzed a comprehensive bibliographic dataset of 4,035 studies to document patterns in GBIF-mediated data use over the past two decades—a time period marked by unprecedented growth in data availability and the advent of modern biodiversity informatics. Past studies on biodiversity databases focused on concerns of their quality (29, 37), biases (23), and gaps (25, 27, 38). A quantitative assessment of biodiversity data use has been lacking, yet such an evaluation is needed to assess the impact of large-scale data mobilization efforts and for the strategic development of data-intensive biodiversity research (22). Our results provide quantitative evidence on the pivotal role of integrated biodiversity data networks to enable research that was previously not possible.

Species occurrence data lie at the heart of macroecology and related fields, so it is perhaps unsurprising that the most common use of GBIF-mediated studies involves species distributions. A similar pattern was reported in a recent review of biodiversity database use (20). However, our topic models allowed finer scale separation of research areas through time; most notably, the varied uses of SDMs. SDMs are a broad class of statistical approaches that estimate species’ potential geographic distributions based on known occurrences and corresponding environmental data (30). We found signs of early shift from initial focus of SDM theory development toward SDM application. In addition to GBIF-mediated data, this trend was undoubtedly driven by the new complementary abiotic datasets that are necessary for such analyses, such as global climate data (e.g., WorldClim) (39) alone was cited in 38% of GBIF-enabled studies and statistical tools (e.g., MaxEnt) (40) was cited in 32% of studies. With >1,000 SDM-related publications per year (41), the field is rapidly developing, including establishing community guidelines for standard reporting to maximize reproducibility that include the citation of GBIF-generated data DOIs (42, 43).

Topic model results illustrated wide-ranging GBIF-mediated research themes. A benefit of automated text-based analytical approaches is that topic classification was not limited to an expected set of research areas. Though not among the most prevalent topics overall, the relative increase in GBIF-mediated research on species interactions is especially noteworthy, indicating research application of taxonomically disparate data. Surprisingly, nearly a 10th of recent GBIF-mediated studies included data from multiple distantly related taxonomic groups (denoted as “>1 category” in Fig. 1*B*). Data use was not directly driven by data availability, as more studies used plant data than expected by proportional representation across GBIF (Fig. 1). Similarly, the increasing research focus on disease is also likely a product of integrating taxa-specific datasets (e.g., invertebrate disease vectors with animal hosts; different trophic levels). As research becomes more cross-disciplinary, taxonomically integrated data should be promoted due to the increasing societal relevance of research on crop and zoonotic disease origins, including intensive taxon sampling needed to pinpoint the origins of SARS-CoV-2 and future threats to human health (44).

Trends in the GBIF-enabled literature calls attention to the value and the need for further integration of otherwise disparate data. A common critique about big data aggregation is a loss of information about the individual datapoints themselves. In addition to extensive natural history collection digitization worldwide (16, 45), the pace at which new observation-based data has been collected through public participation is accelerating. Many are concerned that observation-based occurrences are overshadowing specimens (12), which have long been critical to biodiversity science (46, 47) and providing ecological baselines in a rapidly changing world (45). A somewhat contrary view is that, despite lacking a physical record, the rise of observation-based data collected by humans and machines is necessary to provide large-scale data for large-scale questions (48). Our analysis of GBIF-enabled data use highlights synergistic roles for observation- and specimen-based data when combined. Biodiversity research benefits from both types of data, and the growth of one should not come at the expense of the other.

Beyond connecting data, GBIF bridges research communities by providing the opportunity for synergy between museums, community science efforts, and ecology and evolution fields at large. GBIF’s initial vision was strongly specimen based, as a digital data hub for liberating and accessing the world’s biodiversity data, which at the time was held almost entirely in museums (6). With the development of shared data standards (49) and goals across funding initiatives (16), significant progress has been made toward that vision. Still, only 10 to 20% of specimens are available through GBIF, based on an estimated 1 to 2 billion specimens worldwide (50). Though a sizable increase from 3% just a decade ago, much museum digitization work remains (especially in certain taxa) (51). Museums are becoming increasingly connected through natural history specimen digitization (52) and the integration of complementary data streams (47).

Tremendous effort and financial investments have contributed to GBIF-mediated data. Here, we documented the research made possible by the countless efforts of data collectors, researchers, funding agencies, and data curators. Though we focus on peer-reviewed studies that actively use GBIF-mediated data, this infrastructure has also supported knowledge production and dissemination through other published media. GBIF-mediated data were mentioned or cited in 680 nonpeer-reviewed publications from 2016 to 2019 alone, including student theses, white papers, technical reports, and web pages. Supported by the world’s governments, the growth of the GBIF network required collaborative investments from participant countries and the recognition of the critical value of centralized open access to standardized biodiversity data for the common scientific good. While our results confirm this societal value, the future of data integration must continue toward maximizing global participation to enable a new scientific era that is scientifically and socially inclusive. The open data culture (53) necessitated by this arrangement has contributed to biodiversity knowledge generation by a more globally inclusive and diverse research community (54), including digital repatriation of data to regions with history of exploitation. Although data integration has improved global authorship, research patterns indicate legacies of scientific colonialism persist (Fig. 2), with proportionally more research on the biodiversity of the Global South being authored by researchers in the Global North (*sensu* ref. 53). On one hand, this pattern could be viewed as promising, given that global data integration has enabled researchers from across the world to study biodiversity otherwise not possible. On the other hand, however, nearly 8% of regional studies were completed without regional authors, suggesting needed progress toward mutual international collaboration. Further, the use of data DOIs not only ensures data transparency at the core of open science (53), but also provides a mechanism of data attribution so data providers are aware and recognized for their contributions. However, data citation remains inadequate in biodiversity science, with a recent study finding >33% of papers reviewed provided insufficient citation of biodiversity dataset(s) used and >25% of studies citied databases that were no longer accessible (20).

Our findings inform and validate the prioritization of ongoing and emerging initiatives with common goals to optimize biodiversity science through data integration. As outlined in ref. 16, these include nationally and internationally funded efforts that develop biodiversity data infrastructure, such as, for example, the US National Science Foundation’s Advancing Digitization of Biodiversity Collections (ADBC) program, Australia’s Atlas of Living Australia (ALA), Mexico’s Comisión Nacional Para el Conocimiento y Uso de la Biodiversidad (CONABIO), Brazil’s Sistema de Informação sobre a Biodiversidade Brasileira (SiB-Br) and Centro de Referência em Informação Ambiental (CRIA), and China’s National Specimen Information Infrastructure (NSII). Supported by the European Union, the Distributed System of Scientific Collections (DiSSCo) is actively developing the infrastructure to implement the digital specimen framework for managing the constellation of data related to specimens (55), with synergistic efforts in the United States through the Extended Specimen Network (47). Related efforts include the development of a global registry of the world’s natural history collections (56). These physical, cyber, and human expertise resources are sought to be effectively leveraged together to form an alliance for biodiversity knowledge (57), toward the common goal of biodiversity synthesis.

The far reach of GBIF-mediated data demonstrates biodiversity data integration as both enabling and catalyzing biodiversity science. First, globally integrated datasets enabled researchers to ask questions at taxonomic, temporal, and spatial scales that would otherwise be impossible—for instance, GBIF-mediated studies have enabled cross-taxon global analyses from a global authorship. Second, data integration catalyzes biodiversity research by providing researchers instantaneous data access standardized in a single portal, intensifying the rate at which research can be done—for instance, GBIF-enabled studies have cited use of >26,000 disparate datasets that would otherwise be either unavailable or spread across many databases. Though promising, this work is far from a culmination. Our review highlights the need for continued development to facilitate a new era of data-intensive biodiversity science. We stress the need for continued data digitization and publishing, the creation of data for a more complete unbiased view of biodiversity, efficient routes for providing feedback to improve data quality, new initiatives and tools for linking databases across disparate forms, and a deeper integration of occurrence records with phylogenetic, environmental, phenotypic, ecological, and genetic databases.

## Materials and Methods

### GBIF-Mediated Literature Database.

The GBIF Secretariat curates a long-term, continuously updated bibliographic database by actively tracking the use of GBIF-mediated data in the scientific literature. Possible new GBIF-mediated publications are regularly screened as they are published (*SI Appendix*, Fig. S1), notified through email alerts from journal publishers and literature databases (Google Scholar, Scopus, Wiley Online Library, SpringerLink, NCBI Pubmed, bioRxiv) based on GBIF-related keywords and phrases (e.g., “GBIF,” “Global Biodiversity Information Facility”) and GBIF-assigned dataset DOI prefixes (e.g., 10.15468). Each GBIF-related publication was flagged with a GBIF use category: 1) direct use of data in a quantitative analysis (e.g., species distribution modeling), 2) coarse facts derived from overall data (e.g., species presence in a given country), and 3) mention of GBIF without specific data use. If included, data DOIs were recorded in the database, which is expanded to attribute specific data use to all contributing datasets and data publishers. Bibliographic metadata about each publication was gathered, including type (e.g., journal article, book chapter), countries of author affiliations (including all authors), countries of research coverage (excluding global studies), peer reviewed (yes/no), and open access status at time of publication (yes/no). In the present study, we included all peer-reviewed journal articles that made substantive use of GBIF-mediated data. The final dataset for text analysis (described below) included 4,035 GBIF-mediated peer-reviewed articles with English abstracts, published from 2003 to 2019 (*SI Appendix*, Fig. S1).

### Topic Models.

Automated text analysis was performed on 4,035 articles, including article abstracts, titles, and keywords using the *stm* package (58) in R (59). Publication year was included as a covariate, as we were specifically interested in how topic prevalence changed through time and word usage within topics may vary over time. Although STM is an automated approach, a clear understanding of the analyzed text is required by the user to determine the number of topics to estimate. The “optimal” number of topics modeled depends on prior research on the subject matter, the scope of goals or questions motivating the analysis, and the corpus itself (60). Modeling too few topics lumps otherwise meaningful topics into broad categories that may blur interpretation and modeling too many topics adds superfluous complexity and may result in many topics that lack substantive meaning. Following ref. 60, the decision on the number of topics to model was determined by comparing output from a range of models that differed in number of topics. For each model, a subset of abstracts was read with the highest fractional assignment to each topic to evaluate the thematic cohesiveness of abstracts within each topic and interpretive meaning. Topic model selection and validation is further described in *SI Appendix*.

Topic models have several strengths as tools for identification and mapping of major themes in a body of literature (32). First, manual coding of topics for each paper was unfeasible to do, given the large size and thematic breadth of this bibliographic dataset. Second, as an unsupervised approach, this method avoids potential researcher disciplinary bias or inconsistencies, as manual methods rely on expectations and perspectives of person(s) manually assessing texts. Last, because topic definition is unsupervised, the method allows for the emergence of unexpected research themes, such that topics can be discovered rather than assumed (33).

### Science Map.

To understand the research space of GBIF-mediated studies relative to a broader scientific research landscape, we mapped GBIF-mediated studies onto a widely used reference base map, the University of California San Diego (UCSD) map of science (36) using *Sci2* tool (61). The UCSD map of science was updated in 2010 (36) based on bibliographic analyses to quantify the network of major and minor disciplinary foci (subdisciplines assigned based on journal clustering). Because the UCSD map of science was based in part on the journals indexed in the Web of Science (Clarivate Analytics, formerly ISI), we first exported full bibliographic records from the Web of Science by searching the database via article DOIs in the GBIF-mediated studies on June 4, 2020. This resulted in 3,426 Web of Science records across all years (85% of total GBIF-mediated literature). It is unlikely that the excluded GBIF-mediated studies were a biased subset by research area. Unlike topic modeling, our goal for creating a GBIF map of science was to coarsely visualize actual and potential research use space from a broad perspective (e.g., all journals indexed in the Web of Science). Unlike topic models, journal classifications are indicative of readership, not article level content. We reclassified *PLoS ONE* from its originally assigned single subdiscipline (disease related) to be more accurately interdisciplinary (similar to *PNAS*). This reclassification reduced the proportional representation of GBIF-enabled studies mapped to infectious disease (9 to 3%) but otherwise was similar (*SI Appendix*, Fig. S7). We did not manually assign subdisciplines to unclassified journals to avoid classifications that are inconsistent with existing journal assignments (36).

## Supplementary Material

Supplementary File

## Data Availability

GBIF-mediated literature database (continuously updated) can be found at https://www.gbif.org/resource/search?contentType=literature. Source code and data are available in GitHub at https://github.com/jmheberling/GBIF_Systematic_Review and archived in Zenodo at https://doi.org/10.5281/zenodo.4009481 (62).
