# Supplementary material for: Data integration enables global biodiversity synthesis
Source: Proc Natl Acad Sci U S A. 2021 Feb 1;118(6):e2018093118. doi: 10.1073/pnas.2018093118 (PMC8017944; doi:10.1073/pnas.2018093118)
Supplement: Supplementary File [file pnas.2018093118.sapp.pdf]

PNAS
www.pnas.org

Supplementary Information for
Data integration enables global biodiversity synthesis

J. Mason Heberling[a,1], Joseph T. Miller[b], Daniel Noesgaard[b], Scott B. Weingart[c], Dmitry Schigel[b]
[a]Section of Botany, Carnegie Museum of Natural History, Pittsburgh, PA 15213
[b]Global Biodiversity Information Facility, Secretariat, Universitetsparken 15, DK-2100 Copenhagen Ø, Denmark
[c] Digital Humanities Program, University Libraries, Carnegie Mellon University, Pittsburgh, PA 15213
[1] Corresponding author email: heberlingm@carnegiemnh.org

This PDF file includes:

Appendix S1
Table S1
Figures S1 to S7
SI References

Appendix S1

Additional background on structural topic models. Topic modeling, a form of text analysis often referred to as automated content analysis, broadly refers to a wide set of algorithms and statistical approaches for synthesizing text using machine learning tools (1). Topic models have been widely used in the social sciences, humanities, and medical fields, and more recently applied in ecology and evolutionary biology (2).

Topic models use text-parsing and machine learning tools developed by computer scientists to discover associations between words in large volumes of documents and organize these associations into clusters of words that tend to occur together. These clusters of co-occurring words are the classified topics (sometimes called "concepts", "themes", or "discourses") within a body of literature (corpus). Though the number of topics modeled is chosen by the researcher through a model selection process (see below and Methods in main text), any number of topics can emerge inductively in a corpus (i.e. unsupervised, or not chosen in advance by the researcher) based on word co-occurrence within and between texts and their prevalence across the corpus. To be associated with each other in a topic, words must occur more often in association with each other than they would if drawn at random from the corpus vocabulary, given their individual frequencies in the corpus. Topics are therefore defined by probability distributions for a vocabulary of words that group together more than they group with other word groups. In our review, we used modification of a *latent Dirichlet allocation* (LDA) topic model approach (3). The LDA approach is an unsupervised, mixed-membership model, wherein each word within a document belongs to a given topic and each document can include multiple topics. Each document is represented as a vector of topic proportions according to fractions of words assigned to a given topic. Each topic's importance in the corpus as a whole is a function of proportion of all documents (and words within documents) in the corpus that are associated with the topic.

More specifically, we used an approach called Structural Topic Modeling (STM; (4)), which is derived from the earlier developed *latent Dirichlet allocation* (LDA) topic model approach (3) to quantitatively synthesize trends in GBIF-mediated research through time. As summarized by Roberts et al. (4), these models differ from LDA in that STMs provide "structure" such that: 1) topics can be correlated to each other, providing some insight into relationships among topics and allowing meta-topics to emerge, 2) each document has a prior distribution across topics, defined by covariate(s) instead of a shared global mean; in our study there is a distribution specific to the year of publication of the document, and 3) word prevalence within each topic can vary with a covariate.

Details on the advantages of structural topic models compared to hand coding are briefly described in the main text, and elaborated elsewhere (1, 2, 5). Technical details on structural topic modelling can be found in Roberts et al. (4).

Model validation. As outlined in main text, we followed the model selection procedure described in ref. (5). While structural topic modelling is an unsupervised approach, it still requires user validation in the determination of how many topics to model. Quantitative metrics to facilitate model selection on how many topics to include for a given corpus is through model exclusivity and semantic coherence (4). Semantic coherence measures the frequency that high probability words tend to co-occur in documents, while exclusivity measures the proportion of high probability words that are distinct to a given topic. Theoretically, the "best" model will optimize both of these metrics. However, there is a tradeoff between model exclusivity and semantic coherence. Therefore, in practice, there is not an obvious "best" number of topics to model based on these metrics alone. A comparison of these metrics across many models for the current set of GBIF-mediated study abstracts suggests between 20-30 topics to be most informative (Fig. S2).

In addition to considering model exclusivity and semantic coherence, we closely interpreted the outputs from a range of models with 10-50 topics included. For each model, we read the top 25 abstracts associated with each topic and evaluated the corresponding high probability words that define that topic. This recursive process resulted in the selection of a topic model that provided a comprehensive, yet meaningful, overview of the GBIF mediated literature. Since we had no exact *a priori* prediction on the number of topics that this literature must

encompass, we selected the 25-topic model because the resulting topics were logically sound and covered a diversity of GBIF- and biodiversity-related research areas. This number of topics is also in line with 29 major use topics in a recent manual review of biodiversity database use (6).

Our results were robust to the number of topics modelled and did not qualitatively change the overall conclusions. Many topics were remarkably robust to changes in number of topics modeled, exhibiting the same top abstracts, high probability word sets, and a similar relative rank in prevalence. For example, topics best described as invasion biology, biodiversity informatics, disease, climate futures, and people and nature were consistently present in topic model versions with 20, 25, and 30 topics included. In terms of interpretive value and cohesion among top 25 associated abstracts, other topics were sensitive to the number of topics included in the model. Including fewer topics (Fig. S3) resulted in lumping of otherwise meaningful topics that shared certain words or word relationships (e.g., taxonomic treatments was split, included among two related categories of phylogenetics and novel occurrences), “catch all” topics with less substantial meaning (e.g., disease + species interactions), or topics modified interpretations (e.g., ethnobotany focuses on plants only, where people and nature in 25 topic model was conceptually more inclusive). Similarly, increasing the number of topics (Fig. S4) resulted in additional topics with clear meaning (e.g., crop pests, presumably breaking up what was formerly a disease and invasion species management topics in 25 topic model version), but at the expense of dividing previously meaningful topics into a granularity that was difficult to interpret or not necessary in the context of current study (e.g. taxonomy I, taxonomy II).

Table S1. Structural topic model results from 4,035 studies (including article titles, abstracts, and keywords) using GBIF-mediated data published from 2003 to 2019. Topics are numbered in descending order by proportion of the entire corpus (i.e., all text analyzed), and including topic name, top 15 words with highest probability in each topic, and a general description of each topic. The dominant topic sets of high-probability words are generated by the model while the topic names and general descriptions result from author interpretation based on these words and top abstracts (see Appendix S1 above).

**Topic name**	**Top 15 associated words**	**Topic prevalence** (all years)	**Relative change within topics** (2016-2019 prevalence compared to pre-2016 prevalence)	**General description**
1. Species distribution models (tools/methods/theory)	*model,speci,use,distribut,predict,data,method,estim,spatial,approach,variabl,perform,occurr,map,base*	7%	-10%	Methodological and conceptual studies with main focus on species distribution modeling, including new quantitative approaches, model performance/validation, tests for biases
2. Biodiversity informatics	*data,inform,biodivers,databas,collect,use,avail,research,global,knowledg,provid,dataset,gbif,access,can*	6%	-15%	Information science applied to biodiversity data, including data portals, data creation, georeferencing, and other tools for data use (access, integration, management, visualization, cleaning)
3. Climate futures	*climat,chang,speci,futur,will,impact,rang,scenario,increas,effect,shift,current,project,predict,distribut*	6%	+18%	Prediction of species distributions based on past and projected future climates using quantitative models, often in context of anthropogenic climate change
4. Species distribution models (applications)	*distribut,suitabl,model,potenti,area,habitat,predict,use,climat,current,futur,variabl,temperatur,result,region*	6%	+48%	Applications of species distribution models across scales
5. Novel occurrence/Range extensions	*record,distribut,new,speci,first,report,collect,known,occurr,brazil,present,specimen,local,rang,geograph*	6%	+3%	Reports of unique biodiversity records, especially range extensions and accounts of previously unknown species records
6. Taxonomic treatments	*speci,genus,morpholog,two,new,taxonom,group,taxa,describ,genera,studi,includ,molecular,base,sequenc*	6%	-21%	Taxonomic descriptions and revisions, including new species descriptions, comprehensive treatments of taxon groups (monographs), and taxonomic revisions
7. Conservation	*conserv,area,protect,threaten,speci,biodivers,prioriti,assess,habitat,threat,use,high,identifi,plan,manag*	5%	+25%	Applied conservation studies, including extinction risk assessments, prioritization of biodiversity hotspots, restoration
8. Clade diversification	*phylogenet,clade,lineag,diversif,evolut,evolutionari,origin,dispers,speciat,diverg,phylogeni,use,time,result,histori*	5%	+2%	Phylogeographic studies focused on lineage evolution and speciation
9. Niche dynamics	*nich,rang,ecolog,speci,climat,geograph,distribut,differ,environment,model,overlap,shift,test,result,similar*	5%	-1%	Ecological and evolutionary studies of niches, often also including quantitative niche models within and across species
10. Macroecological diversity patterns	*pattern,speci,rich,divers,region,spatial,scale,environment,differ,use,relationship,variabl,relat,geograph,across*	4%	+6%	Diversity patterns from continental to global scales, often focusing on latitudinal relationships
11. Global invasion dynamics	*america,north,south,island,central,western,asia,northern,eastern,africa,american,rang,southern,region,europ*	4%	-16%	Invasive species spread across continents and through time
12. Functional ecology	*trait,speci,temperatur,seed,toler,adapt,leaf,function,respons,differ,environ,increas,variat,climat,evolut*	4%	-4%	Ecophysiological studies, including intra- and interspecific variation of functional traits and responses across scales and environments

13. Historical biogeography	*distribut,glacial,model,speci,last,pleistocen,phylogeograph,genet,refugia,expans,lgm,pattern,histori,maximum,result*	4%	-18%	Evolutionary studies, including historical effects of paleoclimates and post glacial migration and evolution on present day biodiversity patterns
14. Invasion biology	*invas,speci,nativ,invad,alien,potenti,risk,introduc,spread,establish,introduct,global,region,human,popul*	4%	-9%	Empirical studies of non-native species, including understanding patterns and processes of local spread, invader success, and invasion risk
15. Regional distribution patterns	*speci,endem,distribut,river,mammal,freshwat,studi,amphibian,group,number,region,fish,divers,basin,one*	3%	+15%	Regional distribution patterns of species and taxonomic groups, especially focused on endemism
16. Population genetics	*popul,genet,divers,structur,gene,variat,differenti,flow,geograph,high,among,isol,level,distanc,analys*	3%	-9%	Studies on the genetic structure and diversity of populations across a species' range and environmental conditions
17. People and nature	*use,crop,wild,product,cultiv,resourc,food,medicin,relat,potenti,agricultur,tradit,activ,studi,domest*	3%	-1%	Studies at the interface of biodiversity and human use (ethnobiology), including human health, ethnobotany, conservation of crop wild relatives, and agriculture
18. Marine biology	*marin,sea,ocean,water,atlant,fish,temperatur,arctic,mediterranean,pacif,coastal,coast,coral,gulf,lake*	3%	-11%	Biological studies of marine taxa (in fossil record and extant taxa), including ecology and evolution
19. Forest biology	*forest,tree,habitat,tropic,speci,veget,elev,dri,lowland,mountain,cover,soil,andes,fire,montan*	3%	-1%	Wide-ranging studies involving forest species and communities
20. Phenotype	*size,flower,bodi,differ,reproduct,time,may,phenolog,studi,adult,length,butterfli,nest,male,femal*	2%	+25%	Quantitative studies of organismal phenotypes, often across temporal and spatial scales, including organism size (morphometrics), sex, behavior, and other attributes
21. Spatial ecology	*mexico,area,site,bird,habitat,connect,use,breed,landscap,california,state,ecolog,histor,mexican,speci*	2%	+10%	Range-level of species abundances and their ecology, including migration and breeding patterns
22. Disease	*pest,risk,vector,diseas,human,insect,host,bat,beetl,potenti,virus,health,associ,popul,predat*	2%	+28%	Epidemiology, zoonotic disease vectors, crop pests
23. Invasive species management	*australia,africa,control,south,weed,natur,manag,biolog,zealand,grass,new,garden,australian,current,rang*	2%	-33%	Management of non-native species, including biological, chemical, and manual control agents, as well as biosecurity and prevention efforts
24. Species interactions	*interact,speci,host,pollin,bee,parasit,biotic,isol,studi,declin,orchid,special,network,factor,preval*	2%	+32%	Heterospecific interactions, including non-trophic and trophic interactions such as pollination, predation, parasitism, and herbivory
(Botany*)	*plant,flora,studi,pollen,taxa,vascular,herbarium,divers,mediterranean,soil,communiti,biom,region,alpin,level*	3%	-6%	*Excluded due to low interpretive value (mostly plant-related but little conceptual or methodological connections across top abstracts associated with this topic)

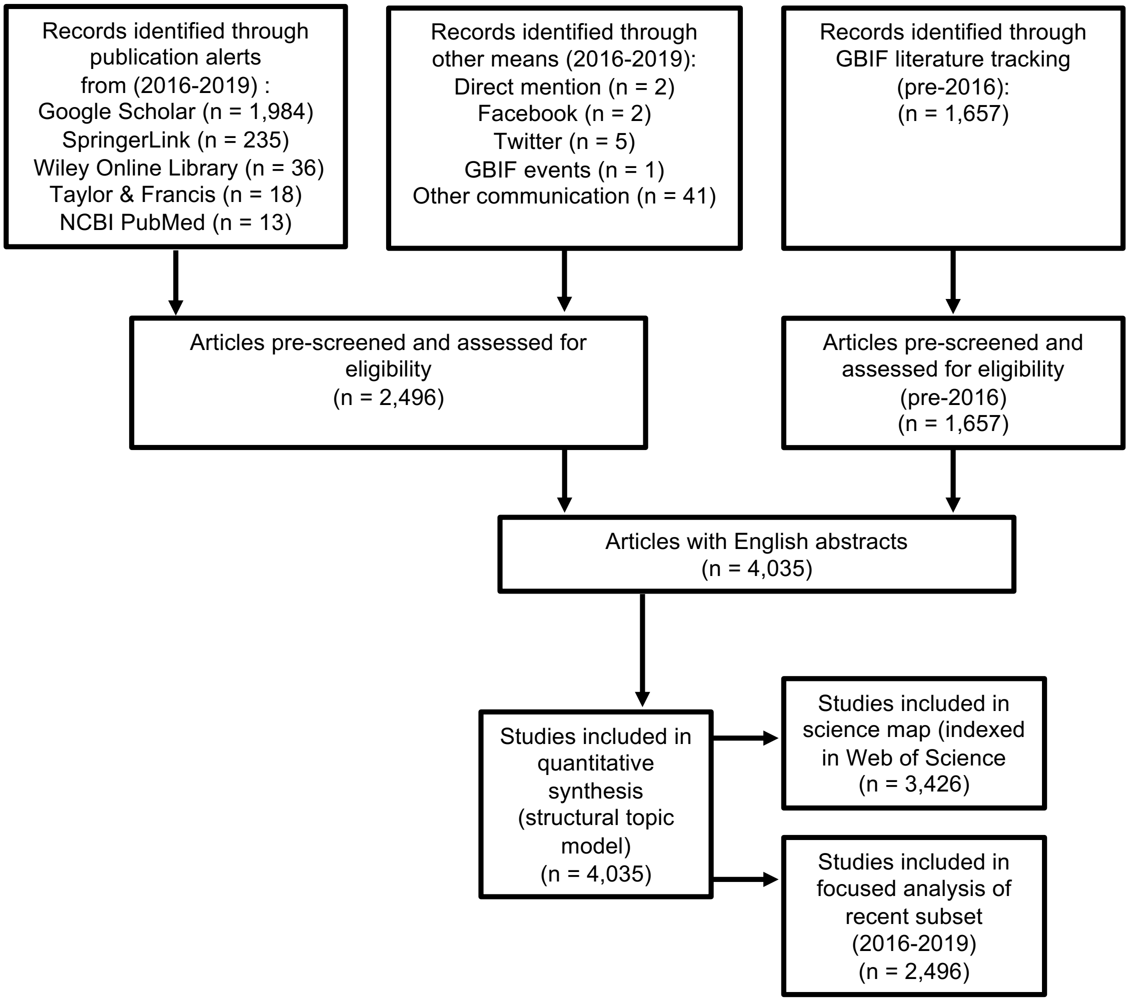

Fig. S1. PRISMA flow chart outlining process of literature compilation for inclusion in bibliometric analysis and topic models.

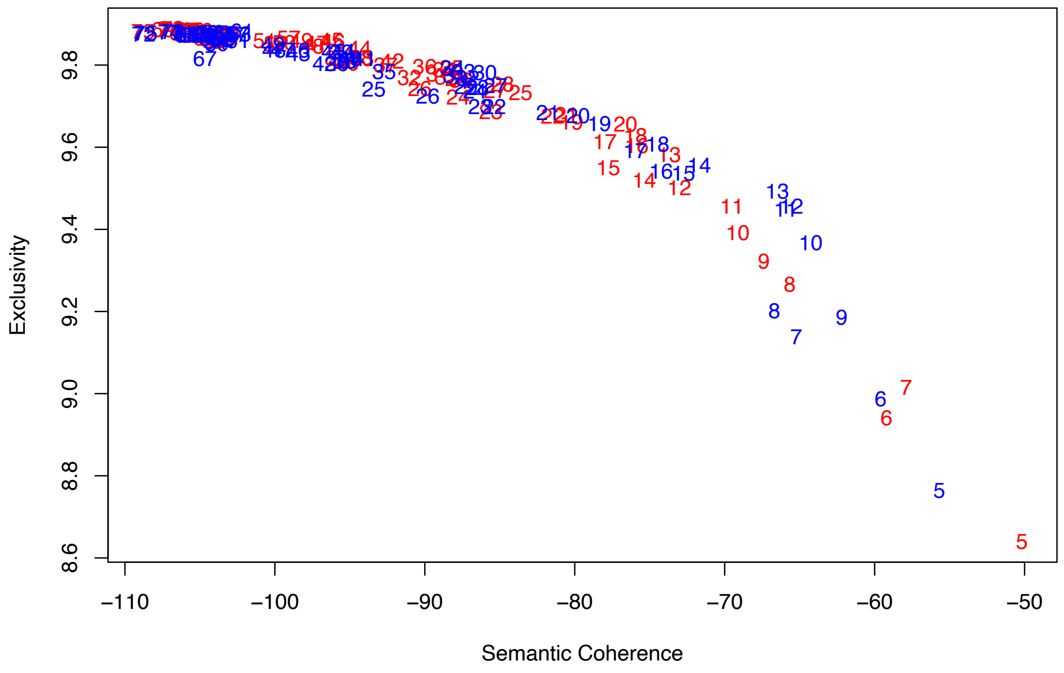

Fig. S2. Relationship between model exclusivity and semantic coherence. Each number refers to a separate topic model that included that number of topics (K). The number's location on the graph corresponds to the average values for that topic model for exclusivity and semantic coherence. Because topic modeling estimation is not deterministic, models were run twice for each number of topics to ensure conclusions were consistent across model runs (model runs denoted by different colors). These model metrics suggest exclusivity and semantic coherence are jointly maximized at around K=20-30. Model presented in main text included 25 topics.

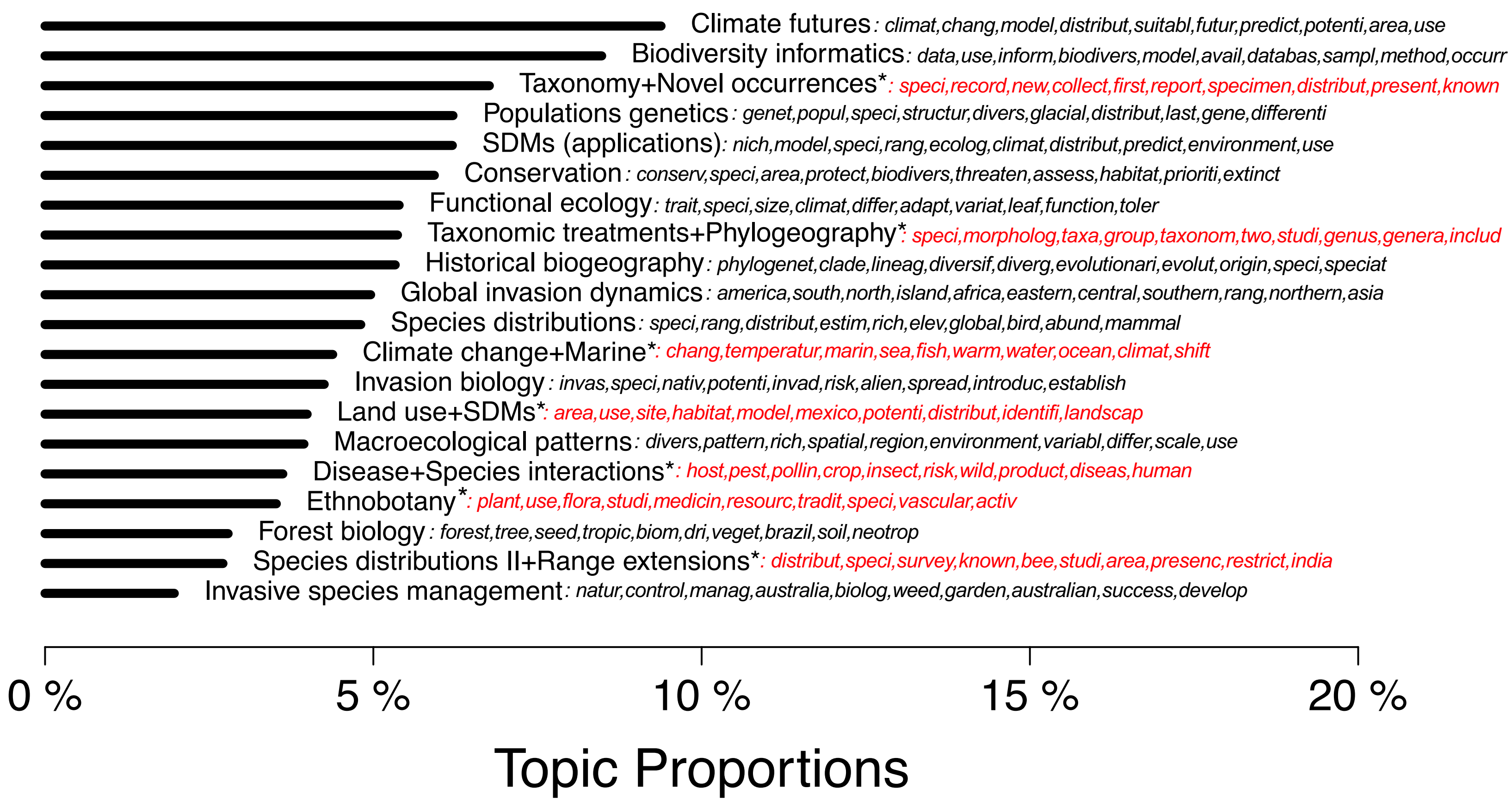

Fig. S3. Topic proportions from 20-topic structural topic model of GBIF-meditated studies. Topic proportions are the percentage of the total corpus classified to each topic. Topic names were defined from top words associated with each topic and holistic themes across the top abstracts related to each topic. The top 10 words associated with each topic are in italics. “Chimera topics” that combine two or more different topics from 25 topic model lack interpretive value or are otherwise different are highlighted in red font and asterisk.

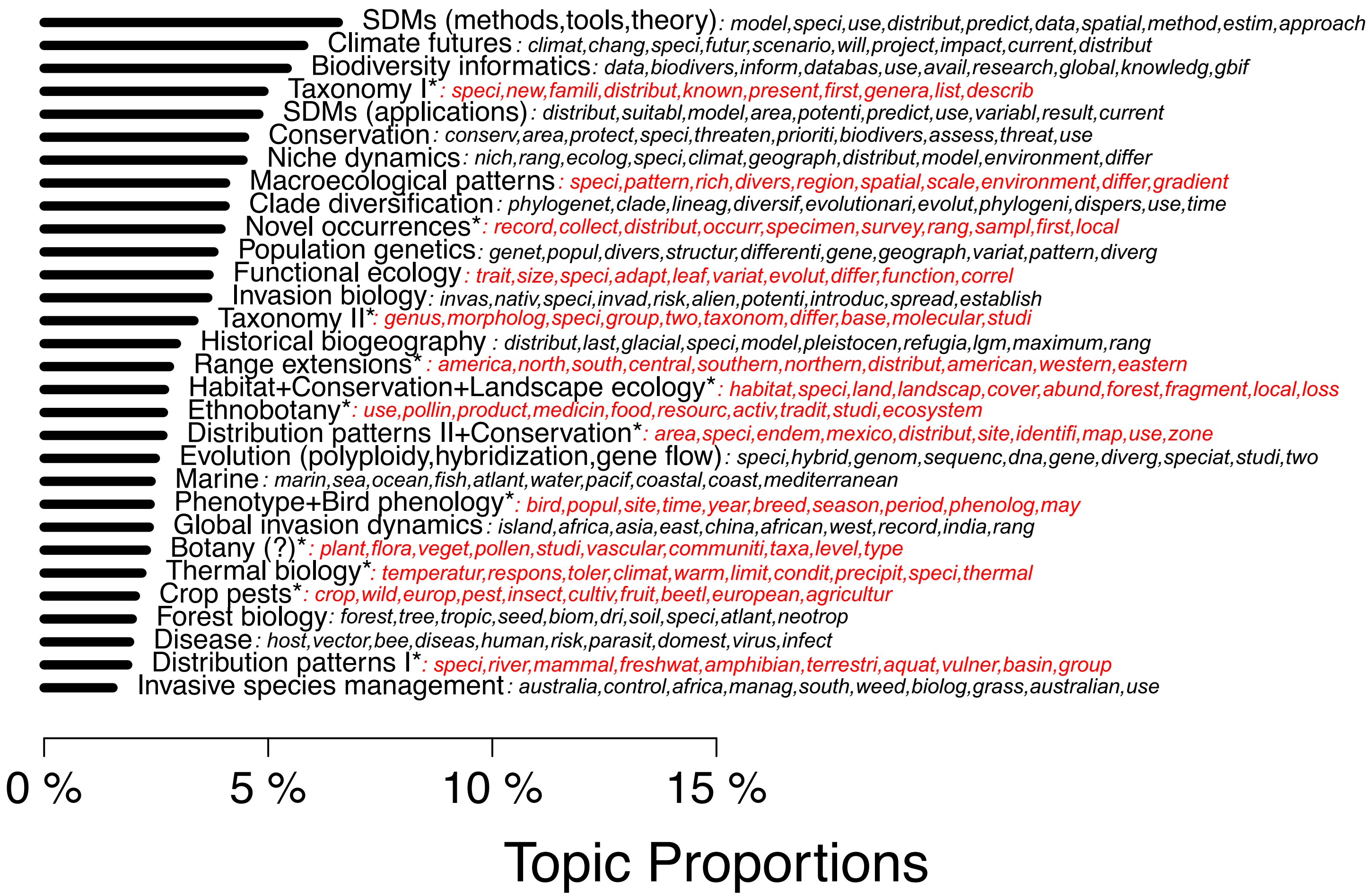

Fig. S4. Topic proportions from 30-topic structural topic model of GBIF-meditated studies. Topic proportions are the percentage of the total corpus classified to each topic. Topic names were defined from top words associated with each topic and holistic themes across the top abstracts related to each topic. The top 10 words associated with each topic are in italics. “Chimera topics” that combine two or more different topics from 25 topic model, lack interpretive value, or are otherwise different are highlighted in red font and asterisk.

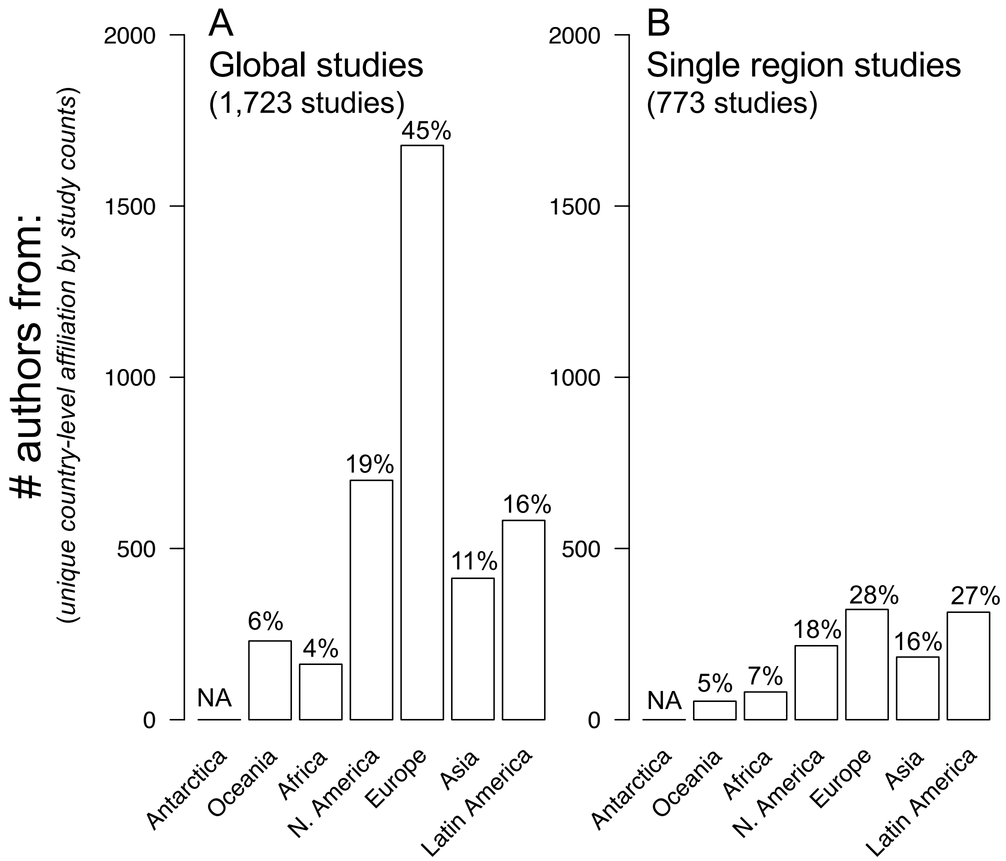

Fig. S5. Authorship affiliation by region from 2,496 GBIF-enabled studies published from 2016-2019 separated by studies of global biodiversity that include biodiversity data from more than one region (*A*) and studies that include biodiversity data from a single region only (*B*). Percentages above each bar denote the proportional breakdown of total unique author affiliation by study counts. Region designations follow ref. (7).

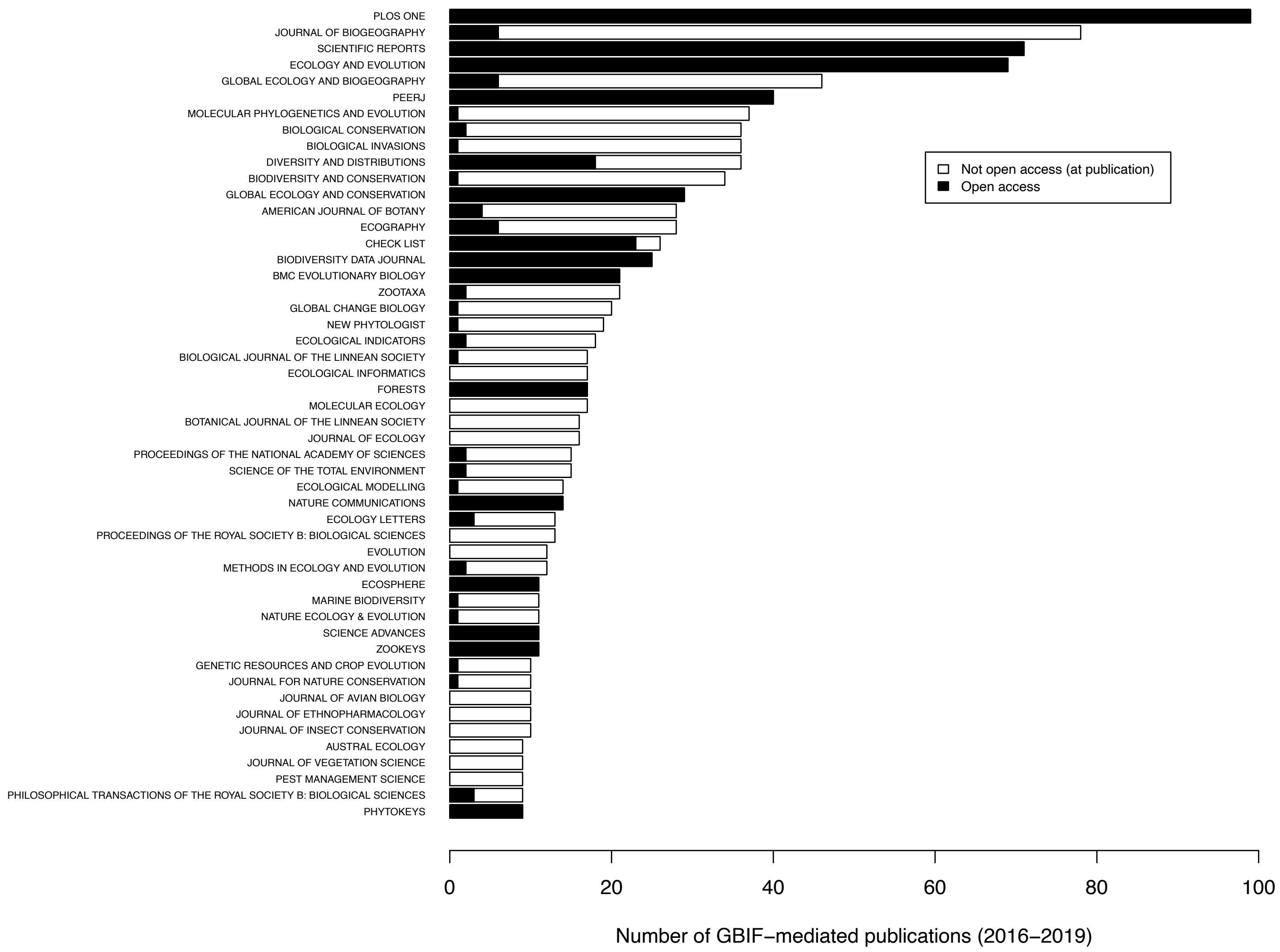

Fig. S6. Number of GBIF-mediated articles published in the 50 most common journals published between 2016 and 2019.

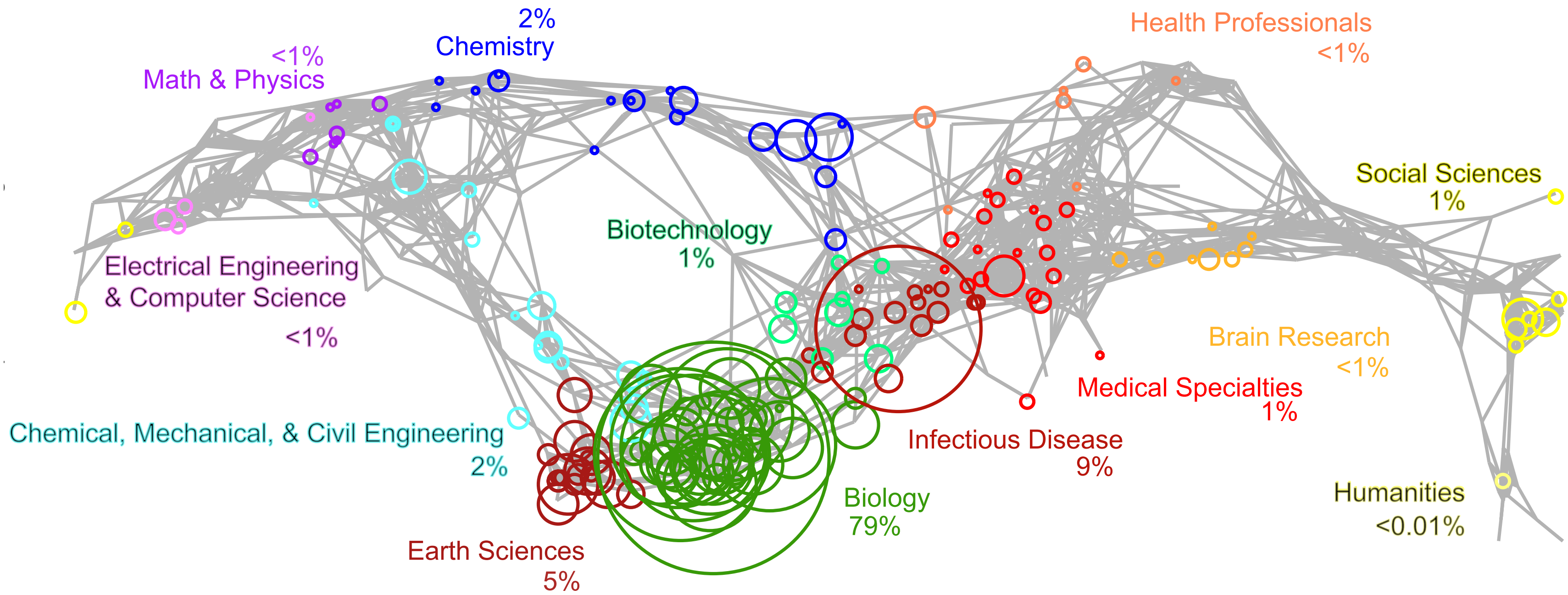

Fig. S7. Uncorrected version of GBIF Map of Science, identical to Fig. 4 except retaining all original journals classifications of the 2010 UCSD Map of Science the UCSD Map of Science (8). As a relatively new journal when the UCSD Map of Science was constructed and has since expanded its primary content areas, *PLoS ONE* was originally classified to a single infectious disease-related subdiscipline through a scientometric analysis based on article content and citation patterns in 2010 (8). Though the journal does publish infectious disease research, we felt it was misleading in this context to classify all GBIF-enabled studies published in *PLoS ONE* under the major discipline of Infectious Disease, especially given the high number of GBIF-enabled studies published in *PLoS ONE* (226 articles). Therefore, we reclassified this journal to be multidisciplinary, with classifications matching *PNAS*, as presented in Fig. 4. This reassignment is suggested by map authors, who decided to map to single subdiscipline for simplicity (8). Note the only major difference is in the Infectious Disease category (9% of studies in uncorrected map, 3% in Fig. 4). As in Fig. 4, this map visualizes the network of interdisciplinary knowledge facilitated through GBIF-mediated data in the context of a broader research landscape. The reference base map (grey lines), the UCSD Map of Science (8), displays a network of >25,000 journals classified across 554 subdisciplines (nodes), grouped into 13 primary disciplines (colors). Circles illustrate GBIF-mediated studies (2003-2019) centered on subdiscipline node assignments with circle size proportion to number of studies. Note that only GBIF-mediated studies published in journals in UCSD Map of Science are included (2,810 articles, 548 journals). Map is a 2D projection of a spherical 3D layout (i.e., the right and left of map connect) and produced using the *Sci2 Tool* (9).

SI References

1. D. M. Blei, Probabilistic topic models. *Commun. ACM* **55**, 77–84 (2012).
2. G. C. Nunez-Mir, B. V. Iannone, B. C. Pijanowski, N. Kong, S. Fei, Automated content analysis: Addressing the big literature challenge in ecology and evolution. *Methods Ecol. Evol.* **7**, 1262–1272 (2016).
3. D. M. Blei, A. Y. Ng, M. I. Jordan, Latent dirichlet allocation. *J. Mach. Learn. Res.* **3**, 993–1022 (2003).
4. M. E. Roberts, *et al.*, Structural topic models for open-ended survey responses. *Am. J. Pol. Sci.* **58**, 1064–1082 (2014).
5. J. Farrell, Corporate funding and ideological polarization about climate change. *Proc. Natl. Acad. Sci.* **113**, 92–97 (2016).
6. J. E. Ball-Damerow, *et al.*, Research applications of primary biodiversity databases in the digital age. *PLoS One* **14**, e0215794 (2019).
7. T. M. Brooks, *et al.*, Analysing biodiversity and conservation knowledge products to support regional environmental assessments. *Sci. Data* **3**, 160007 (2016).
8. K. Börner, *et al.*, Design and update of a classification system: The UCSD map of science. *PLoS One* **7**, e39464 (2012).
9. Sci2 Team, Science of Science Tool (Sci2). Indiana University and SciTech Strategies, Version 1.3. https://sci2.cns.iu.edu/user/index.php. (2018). Accessed 2 July 2020.
